# Medicaid Managed Care Naloxone Coverage and Management

**DOI:** 10.1001/jamanetworkopen.2025.12866

**Published:** 2025-05-29

**Authors:** Sage R. Feltus, Jeffrey Bratberg, Sophia Balkovski, Maureen T. Stewart

**Affiliations:** 1 Department of Health Law, Policy and Management, Boston University School of Public Health, Boston, Massachusetts; 2 Department of Pharmacy Practice and Clinical Research, University of Rhode Island College of Pharmacy, Kingston; 3 Institute for Behavioral Health, The Heller School for Social Policy and Management, Brandeis University, Waltham, Massachusetts

## Abstract

This cross-sectional study evaluates naloxone coverage and management for Medicaid enrollees in managed care plans.

## Introduction

Naloxone, an essential medication for preventing fatal opioid overdose, became available over the counter in 2023; however, cost may still be a barrier to access.^1^ Insurance coverage is a sustainable strategy for low- and no-cost naloxone.^2^ Medicaid provides health insurance for 82 million low-income people,^3^ and 80% of enrollees are in managed care plans (MCPs). The opioid overdose crisis disproportionately impacts Medicaid enrollees,^4,5^ and paying for naloxone out of pocket may not be feasible for many people. Despite this, little is known about naloxone coverage within MCPs. This cross-sectional study systematically examined naloxone coverage and management for Medicaid enrollees in MCPs.

## Methods

In this cross-sectional study, comprehensive MCPs serving enrollees aged 18 to 64 years in 2023 were identified from state Medicaid websites. Preferred drug lists (PDL) for medication coverage and management were obtained from MCP websites or state websites in cases of state-defined PDLs. PDLs are used to negotiate rebates with drug manufacturers and facilitate access to cost-effective medications; some states take this responsibility while others leave it to MCPs. PDLs were reviewed for coverage and management of all available brand and generic intranasal (4-mg), generic injectable and high-dose intranasal (8-mg) naloxone formulations. Whether PDLs required an opioid use disorder (OUD) diagnosis, employed quantity limits (ie, caps on the amount covered per fill) or required prior authorization was documented. State opioid overdose death rates in 2023 were obtained from KFF (low was defined as <75th percentile or <32.5% and high was defined as ≥32.5%).^6^ This study followed Strengthening the Reporting of Observational Studies in Epidemiology (STROBE) reporting guidelines and was exempt from institutional review board approval and the need for informed consent in accordance with 45 CFR §46 because it was not human participant research.

Descriptive statistics examined naloxone coverage and management overall by state, and χ^2^ tests examined associations of coverage with use of a state-defined PDL and state opioid overdose death rates. A 2-sided *P *value less than .05 was statistically significant. Data were analyzed using Stata, version 18.0 from November to December 2024.

## Results

Among all 264 comprehensive MCPs in 40 states and Washington, D.C. in 2023 covering 65.3 million enrollees, most PDLs included generic injectable (242 MCPs [91.7%]), generic intranasal (230 MCPs [87.1%]), brand intranasal (186 MCPs [70.5%]), and high-dose naloxone (179 MCPs [67.8%]) (Table). Quantity limits varied by product. OUD diagnosis or prior authorization requirements were rare. A total of 249 MCPs (94.3%) covered at least 1 generic injectable or 4-mg generic or brand intranasal naloxone formulation, and 241 (91.3%) covered at least one 4-mg brand or generic intranasal and the injectable formulation. In 20 states, all MCPs covered at least 1 generic injectable and generic or brand intranasal formulation without any restrictions (Figure).

**Table.** Medicaid Managed Care Plan (MCP) Coverage and Utilization Management of Naloxone Formulation, 2023

Coverage and policies	MCPs, No (%)^a^				Enrollees, No (%)			
	Generic injectable	Generic intranasal (4 mg)	Brand (4 mg)	High dose (8 mg)	Generic injectable	Generic intranasal (4 mg)	Brand (4 mg)	High dose (8 mg)
Coverage								
Overall	242 (91.7)	230 (87.1)	186 (70.5)	179 (67.8)	59.2 M (90.7)	56.9 M (87.1)	40.9 M (62.6)	38.7 M (59.3)
Used state PDL	130 (90.3)^b^	119 (91.5)	104 (72.2)	103 (71.5)	40.6 M (90.4)	38.1 M (84.9)	25.3 M (56.4)	25.3 M (56.4)
Did not use state PDL	112 (93.3)^b^	111 (92.5)	82 (68.3)	76 (63.3)	18.6 M (91.2)	18.8 M (92.2)	15.6 M (76.5)	13.4 M (65.7)
Low state overdose death rate	191 (98.0)^c^	177 (92.7)^c^	147 (75.4)^c^	145 (74.3)^c^	49.9 M (97.3)	47.3 M (92.2)	33.4 M (65.1)	32.0 M (62.4)
High state overdose death rate	51 (73.9)^c^	53 (76.8)^c^	39 (56.5)^c^	34 (49.3)^c^	9.2 M (65.9)	9.6 M (68.2)	7.5 M (53.7)	6.7 M (47.7)
OUD diagnosis required								
Overall	8 (3.3)	8 (3.5)	20 (10.8)	7 (3.9)	1.8 M (2.8)	1.9 M (2.8)	7.2 M (11.1)	1.8 M (2.8)
Used state PDL	4 (2.8)	6 (4.2)	18 (12.5)	6 (4.2)	914 601 (2.0)	1.5 M (3.4)	6.9 M (15.3)	1.5 M (3.4)
Did not use state PDL	4 (3.3)	2 (1.7)	2 (1.7)	1 (0.8)	917 685 (4.5)	346 711 (1.7)	346 711 (1.7)	315 185 (1.6)
Low state overdose death rate	6 (3.1)	8 (4.1)	20 (10.3)^b^	7 (3.6)	1.3 M (2.5)	1.9 M (3.6)	7.2 M (14.1)	1.8 M (3.5)
High state overdose death rate	2 (2.9)	0	0^b^	0	570 974 (4.1)	0	0	0
Employed quantity limits								
Overall	40 (16.5)	49 (21.3)	37 (19.9)	41 (22.9)	7.9 M (12.1)	9.6 M (14.7)	7.5 M (11.5)	7.7 M (11.7)
Used state PDL	7 (4.9)^b^	7 (4.9)^b^	8 (5.6)^b^	6 (4.2)^b^	1.6 M (3.6)	1.6 M (3.6)	1.8 M (3.9)	1.5 M (3.4)
Did not use state PDL	33 (27.5)^b^	42 (35.0)^b^	29 (24.2)^b^	35 (29.2)^b^	6.3 M (30.7)	8.0 M (39.3)	5.8 M (28.3)	6.1 M (30.0)
Low state overdose death rate	23 (11.8)^c^	29 (14.9)^c^	23 (11.8)^c^	24 (12.3)^c^	4.5 M (8.7)	5.9 M (11.6)	4.9 M (9.5)	4.3 M (8.4)
High state overdose death rate	17 (24.6)^c^	20 (29.0)^c^	14 (20.3)^c^	17 (24.6)^c^	3.4 M (24.4)	3.7 M (26.2)	2.7 M (19.1)	3.3 M (23.9)
Required prior authorization								
Overall	0	9 (3.9)	9 (4.8)	24 (13.4)	0	1.2 M (1.9)	2.5 M (3.8)	3.9 M (6.1)
Used state PDL	0	4 (2.8)	3 (2.1)	12 (8.3)	0	262 406 (0.6)	1.3 M (2.9)	1.9 M (4.3)
Did not use state PDL	0	5 (4.2)	6 (5.0)	12 (10.0)	0	962 868 (4.7)	1.2 M (5.9)	2.0 M (10.0)
Low state overdose death rate	0	9 (4.6)	3 (1.5)^c^	23 (11.8)	0	1.2 M (2.4)	518 035 (1.0)	3.9 M (7.6)
High state overdose death rate	0	0	6 (8.7)^c^	1 (1.4)	0	0	1.9 M (14.1)	96 636 (0.7)

Abbreviations: M, million; PDL, preferred drug list.

^a^ % Represents the row percentage (ie, percentage of plans subject to a state PDL that cover generic injectable and percentage of Medicaid managed care plan enrollees in plans subject to a state PDL that covers generic injectable). A low overdose death rate represents a state opioid overdose death rate lower than the 75th percentile (32.5%) of states included in analyses.

^b^ A significant association by use of state-defined PDL at *P* < .05.

^c^ A significant association by state overdose death rate at *P* < .05.

**Figure.**
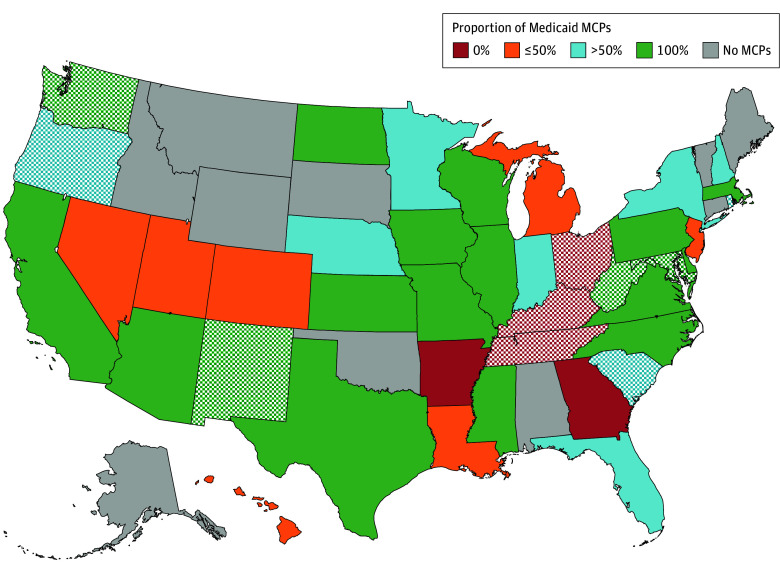
Proportion of Medicaid Managed Care Plans (MCP) in State Covering at Least 1 Generic Injectable or Generic or Brand Intranasal Naloxone Formulation Without Any Restrictions The crosshatch pattern indicates the state had an opioid overdose death rate per 100 000 people in the 75th percentile or above (32.5%).

Bivariate analyses revealed 144 MCPs (54.5%) covering 44.9 million enrollees were subject to a state-defined PDL and 69 (26.1%) covering 14 million enrollees operated in states with high opioid overdose mortality rates. Plans subject to a state-defined PDL were less likely to cover generic injectables and less likely to use quantity limits on any formulation. MCPs in states with low state overdose rates were more likely to cover all forms of naloxone and less likely to require quantity limits.

## Discussion

In this cross-sectional study, almost all Medicaid MCPs covered at least 1 formulation of naloxone; restrictions varied by state. Overdose deaths are disproportionately high among Medicaid enrollees.^4^ Three states with no MCPs covering at least 1 formulation of naloxone without restrictions had high overdose death rates in 2023.^6^ This association is not casual and could be due to many factors; further research is needed. States and MCPs can enhance coverage and limit restrictions on naloxone by updating PDLs. Additional research is needed on how variation in coverage affects enrollees.

Some MCPs may cover naloxone but not report it in the PDL; this is a limitation of our study. Publicly accessible, consistent, and comprehensive information on naloxone coverage and management could help clinicians and patients understand benefits.

## Notes

Supplement.
Data Sharing Statement
