# Supplementary material for: Medicaid Managed Care Naloxone Coverage and Management
Source: JAMA Netw Open. 2025 May 29;8(5):e2512866. doi: 10.1001/jamanetworkopen.2025.12866 (PMC12123468; doi:10.1001/jamanetworkopen.2025.12866)
Supplement: Supplement. — Data Sharing Statement [file jamanetwopen-e2512866-s001.pdf]

Data Sharing Statement

Feltus. Medicaid Managed Care Naloxone Coverage and Management. *JAMA Netw Open*. Published May 29, 2025. doi:10.1001/jamanetworkopen.2025.12866

Data
Data available: Yes
Data types: Data (not involving human participants)
How to access data: Data will be made available upon request. Please contact Dr. Maureen Stewart at stewartm@bu.edu.
When available: With publication

Supporting Documents
Document types: None

Additional Information
Who can access the data: Researchers whose proposed use of the data has been approved.
Types of analyses: The data will be made available for analyses reviewed by the study principal investigator (Stewart).
Mechanisms of data availability: After approval of a proposal and with a signed data access agreement.
